# Reduced Parasite Motility and Micronemal Protein Secretion by a p38 MAPK Inhibitor Leads to a Severe Impairment of Cell Invasion by the Apicomplexan Parasite *Eimeria tenella*

**DOI:** 10.1371/journal.pone.0116509

**Published:** 2015-02-17

**Authors:** Françoise I. Bussière, Fabien Brossier, Yves Le Vern, Alisson Niepceron, Anne Silvestre, Thibaut de Sablet, Sonia Lacroix-Lamandé, Fabrice Laurent

**Affiliations:** 1 Apicomplexes et Immunité Mucosale, INRA, UMR1282, Infectiologie et Santé Publique, F-37380 Nouzilly, France; 2 Université François Rabelais de Tours, UMR1282, Infectiologie et Santé Publique, F-37000 Tours, France; 3 Plate-forme d’Analyse Intégrative des Biomolécules, Laboratoire de Cytométrie et Fluorimétrie, INRA, UMR1282, Infectiologie et Santé Publique, F-37380 Nouzilly, France; Oswaldo Cruz Institute (IOC-Fiocruz), BRAZIL

## Abstract

*E*. *tenella* infection is associated with a severe intestinal disease leading to high economic losses in poultry industry. Mitogen activated protein kinases (MAPKs) are implicated in early response to infection and are divided in three pathways: p38, extracellular signal-regulated protein kinase (ERK) and c-Jun N-terminal kinase (JNK). Our objective was to determine the importance of these kinases on cell invasion by *E*. *tenella*. We evaluated the effect of specific inhibitors (ERK: PD98059, JNKII: SP600125, p38 MAPK: SB203580) on the invasion of epithelial cells. Incubation of SP600125 and SB203580 with epithelial cells and parasites significantly inhibited cell invasion with the highest degree of inhibition (90%) for SB203580. Silencing of the host p38α MAPK expression by siRNA led to only 20% decrease in cell invasion. In addition, when mammalian epithelial cells were pre-treated with SB203580, and washed prior infection, a 30% decrease in cell invasion was observed. This decrease was overcome when a p38 MAPK activator, anisomycin was added during infection. This suggests an active but limited role of the host p38 MAPK in this process. We next determined whether SB203580 has a direct effect on the parasite. Indeed, parasite motility and secretion of micronemal proteins (EtMIC1, 2, 3 and 5) that are involved in cell invasion were both decreased in the presence of the inhibitor. After chasing the inhibitor, parasite motility and secretion of micronemal proteins were restored and subsequently cell invasion. SB203580 inhibits cell invasion by acting partly on the host cell and mainly on the parasite.

## Introduction

The *Eimeria* genus belongs to the Apicomplexa *phylum* and is composed of obligate intracellular parasites that colonize intestinal epithelium causing coccidiosis, a disease that leads to high economic losses in poultry industry [1]. Within the seven species of *Eimeria* that infect chicken, *Eimeria tenella (E*. *tenella)* is one of the most virulent [2] that can lead to death in severe infections. The intensive use of drugs to control the disease led to parasite resistance against all anticoccidial drugs (reviewed in [3]). Therefore, the need for the development of new control strategies against coccidiosis requires a better understanding of the interaction between the parasite and its host.

Invasion of epithelial cells by Apicomplexa is an active process that involves sporozoite gliding motility and formation of a moving junction implicating parasite specialized secretory organelles, the rhoptries of the neck (RON) and micronemes as well as a variety of host receptors [4–7]. Secretion of micronemal proteins occurs rapidly when parasites are in contact with host cells and are found before invasion onto the surface of both parasite and host cell [4,8–11]. When micronemal protein expression or secretion is altered by either inhibitory antibodies [12–15] or chemicals [10,16], cell invasion is inhibited. Micronemal proteins are therefore attractive targets for chemotherapy against Apicomplexa.

Protein kinases constitute one of the largest “superfamilies” of eukaryotic proteins and play many key roles in biology and diseases. Kinases are known to phosphorylate substrates leading to the regulation of major mechanisms including proliferation, gene expression, metabolism, motility, membrane transport, and apoptosis (reviewed in [17]). In mammalians, three major groups of MAP kinases have been described: p38, extracellular signal-regulated protein kinase (ERK) and c-Jun N-terminal kinase (JNK). In Apicomplexa infections, inhibition of MAPK have been shown to decrease host cell infection [18–23] leading to an increase host survival [18]. Studies using p38 MAPK inhibitors attributed this decrease in parasite burden to a lower parasite replication [18,19,23]. Other studies performed with *Toxoplasma gondii (T*. *gondii)* showed that inhibitors of ERK and p38 MAPK pathways, led to a decrease in cell invasion [20,22] but the mechanism has not been identified.

Here, we investigated, the implication of MAPK in host epithelial cell invasion using various cell lines and inhibitors during the infection with *E*. *tenella*. We report for the first time that both the inhibitors of JNKII and p38 MAPK pathways block cell invasion by *E*. *tenella*, with the strongest effect attributed to the p38 MAPK inhibitor, SB203580. This impairment of cell invasion is mainly due to effects on the parasite as shown by alteration of *E*. *tenella* gliding motility and micronemal protein secretion and, to a lower extent, on the host cell p38 MAPK. Therefore, targeting parasite kinases involved in expression or secretion of functional micronemal proteins may lead to the development of a novel generation of anticoccidial drugs.

## Results

### JNKII and p38 MAPK inhibitors decrease epithelial cell invasion in a dose-dependent manner

**Fig 1:**
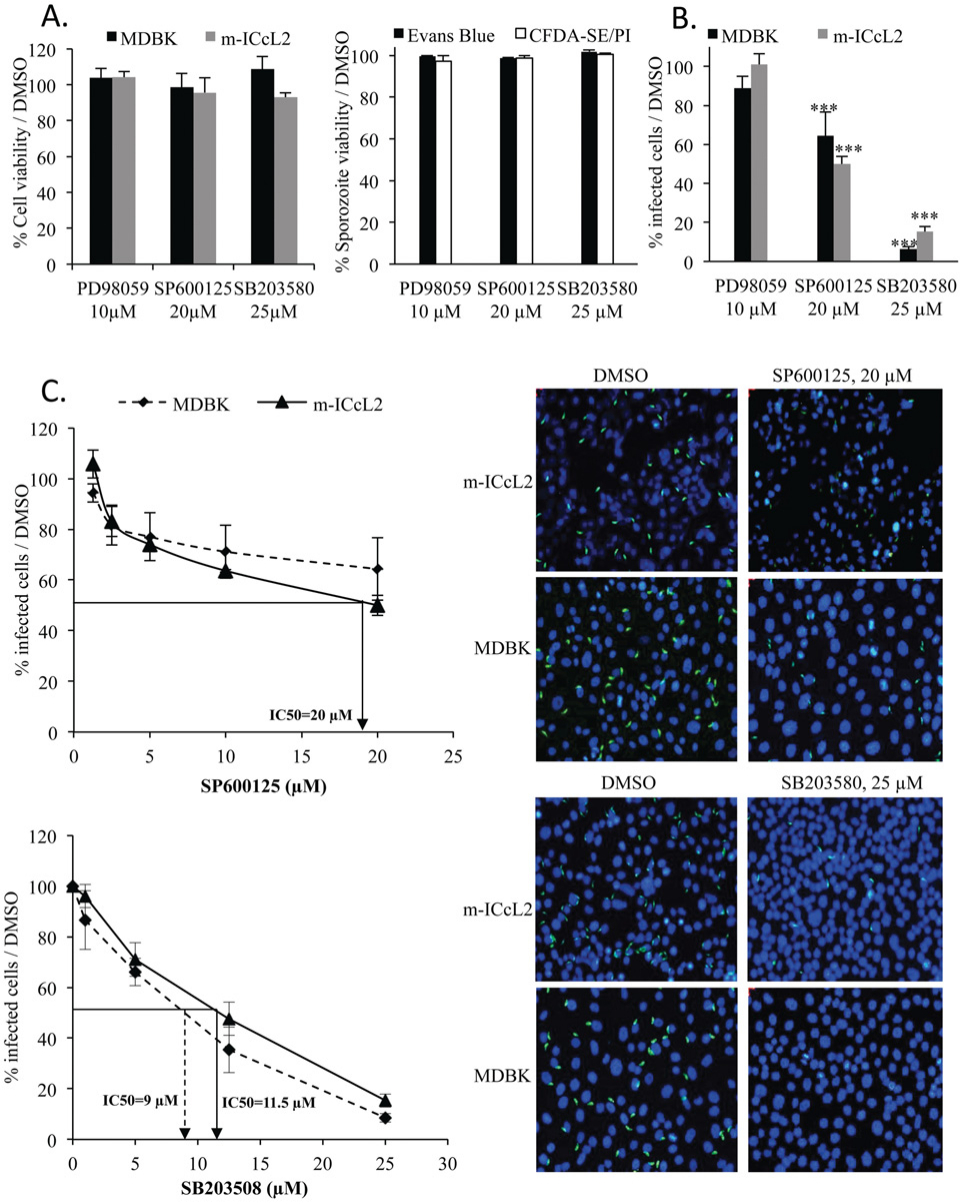
Inhibitors of JNKII and p38 MAPK pathways decrease epithelial cell invasion by *E*. *tenella*. (**A**) Evaluation of the toxicity of kinase inhibitors of ERK (PD98059; 10 μM), JNKII (SP600125; 20 μM) or p38 MAPK (SB203580; 25 μM) on epithelial cells and sporozoites. Percentage of viable cells and sporozoites is determined when incubated with inhibitors and compared to DMSO. *Left panel*: Epithelial cells (MDBK, m-ICcL2) viability was measured by MTT after 2 h incubation with inhibitors. *Right panel*: Sporozoite viability was assessed by Evans blue and CFDA-SE/PI staining after 2 h incubation with inhibitors. (**B**) Evaluation of kinase inhibitors on epithelial cell invasion. Cell invasion by *E*. *tenella* sporozoites was determined after 2 h infection and expressed as the percentage of infected cells in the presence of inhibitors compared to DMSO-treated cells. (**C**) Dose response curve of JNKII and p38 MAPK inhibitors on epithelial cell invasion. *Upper left panel*: Effect of the JNKII inhibitor, SP600125 on epithelial cell invasion. *Upper right panel*: Representative picture of epithelial cell invasion in the presence of SP600125 (20 μM) or DMSO. *Lower left panel*: Effect of the p38 MAPK inhibitor, SB203580 on epithelial cell invasion. *Lower right panel*: Representative picture of epithelial cell invasion in the presence of SB203580 (25 μM) or DMSO. Data represent the mean of 2–3 independent experiments ± SEM. Statistical significance was calculated using the Student t test: *P < 0.05; ** *P* < 0.01 and *** *P* < 0.001.

Since kinases are implicated in major cellular pathways in infection [17,24], we determined the effect of inhibitors of ERK (PD98059), JNK (SP600125) and p38 MAPK (SB203580) pathways on epithelial cell invasion by the apicomplexan parasite *E*. *tenella*. The toxicity of the inhibitors was first assessed on MDBK and m-ICcL2 epithelial cell lines by MTT and on parasites by Evans blue exclusion and CFDA-SE-PI assays. Concentrations of the inhibitors were chosen according to the literature. We first confirmed, in our experimental conditions, the non toxicity of inhibitors both on cells and parasites (> 95% viability) (Fig. 1A and S1 Fig.). We next added inhibitors concomitantly to sporozoites and cells; the percentage of infected epithelial cells was calculated after 2 h infection compared to DMSO conditions (Fig. 1). In our experimental conditions, a low number of cells were infected by more than one sporozoite. In these conditions, the inhibition of ERK pathway by PD98059 (10 μM) did not modify cell invasion by *E*. *tenella* suggesting that kinases from this pathway or parasite homologues are not involved in cell invasion. At 20 μM, JNKII inhibitor, SP600125 led to a 35% and 50% decrease in the number of infected cells while at 25 μM, the inhibitor of p38 MAPK, SB203580 drastically decreased the percentage of infected cells by 91% and 85% in MDBK and m-ICcL2, respectively (Fig. 1B and Fig. 1C (images)). A dose dependent decrease in the number of infected cells occurred both in the presence of SP600125 or SB203580. The IC50 value of SP600125 was close to the highest non-toxic concentration and was defined to be 20 μM for m-ICcL2 (Fig. 1C, *upper left panel*). The IC50 values of SB203580 were determined to be 9 and 11.5 μM for MDBK and m-ICcL2, respectively (Fig. 1C, *lower left panel*). Unfortunately, there is no chicken intestinal epithelial cell line available. However, we had the opportunity to use chicken lung epithelial cells (CLEC-213; [25]) which confirmed the decrease in cell invasion in the presence of SB203580 at concentrations that were not toxic for the epithelial cells; the IC50 was found to be 23 μM (S2 Fig. panels A and B). As the p38 MAPK inhibitor presented the stronger inhibitory effect on cell invasion, we focused on this inhibitor for the rest of the study.

### The host p38 MAPK plays a partial role on cell invasion by *E*. *tenella*

**Fig 2:**
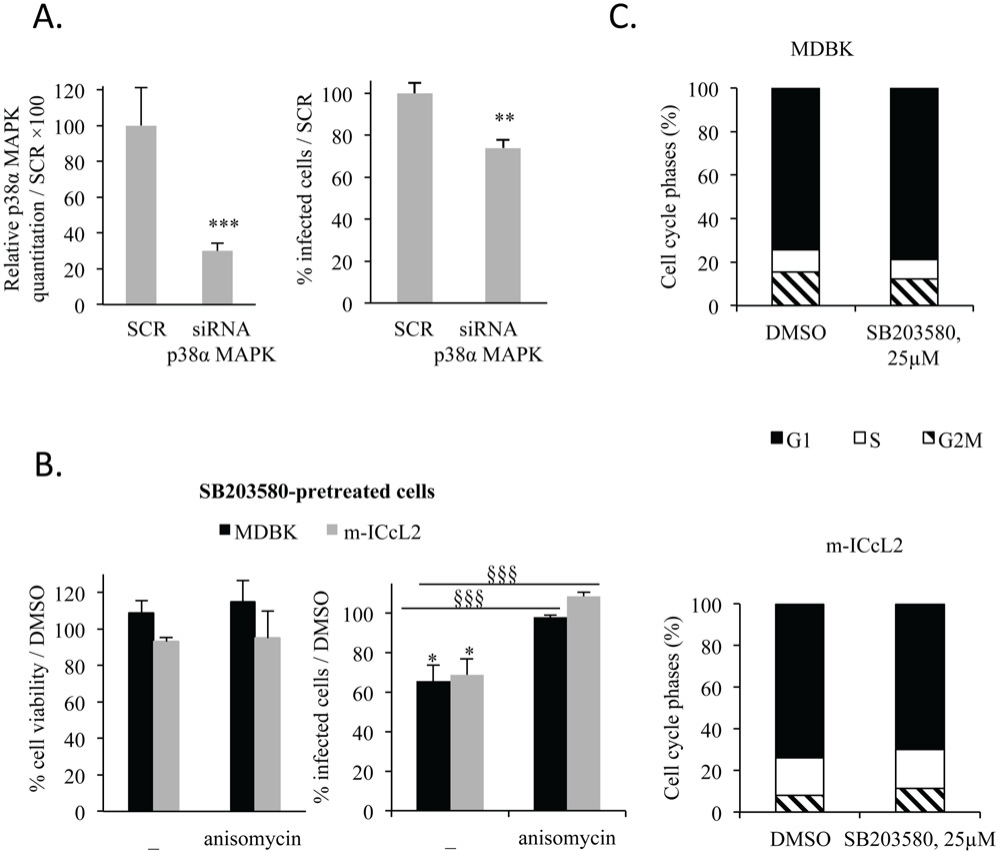
Specific inhibition of host p38α MAPK leads to a minor decrease in epithelial cell invasion. (**A**) Effect of specific inhibition of the host p38α MAPK by siRNA on cell invasion. m-ICcL2 were transfected with either scramble (SCR) or p38α MAPK siRNA. 24 h post-transfection, epithelial cells were infected for 2 h with sporozoites. *Left panel*: Gene expression of p38α MAPK was assessed by RT-qPCR. *Right panel*: Epithelial cell invasion was evaluated and expressed as a percentage of infected cells transfected with p38α MAPK siRNA compared to scramble. (**B**) Effect of SB203580 pre-treatment of epithelial cell (MDBK and m-ICcL2) on cell invasion. Pre-treatment: epithelial cells were incubated overnight with either SB203580 (25 μM) or DMSO. After pre-treatment, cells were washed and treated or not with anisomycin (1 μg/ml) or DMSO during the infection. *Left panel*: Epithelial cell (MDBK, m-ICcL2) viability was measured by MTT. Data are represented as percentage of viable cells compared to DMSO pre-treated cells. *Right panel*: Cell invasion is represented as percentage of infected cells compared to DMSO-treated or DMSO pre-treated cells. (**C**) Effect of SB203580 on epithelial cell cycle. MDBK and m-ICcL2 were treated overnight with SB203580 (25 μM) or DMSO. After washing, cells were fixed, stained with propidium iodide and the epithelial cell cycle was assessed by flow cytometry. Data represent the mean of 3–6 independent experiments ± SEM. Statistical significance was calculated using the Student t test: *P < 0.05; ** *P* < 0.01 and *** *P* < 0.001 when compared to control (SCR or DMSO); ^§§§^ *P* < 0.001 when compared to SB203580 pre-treated cells.

The decrease in cell invasion in the presence of the inhibitor SB203580 could result from inhibition of host p38 MAPK, *E*. *tenella* p38 MAPK homologues or both. Therefore, to study the implication of host p38 MAPK in cell invasion by *E*. *tenella*, we transfected epithelial cells with a mixture of siRNA duplex specific for p38α MAPK or scrambled control siRNA. Transfection of m-ICcL2 with p38α MAPK siRNA caused a significant reduction (70%) in p38α MAPK gene expression (Fig. 2A, *left panel*). The decrease in p38α MAPK gene expression is concomitant to a small but significant (20%) decrease in the number of infected cells transfected with p38α MAPK siRNA compared to cells transfected with scramble siRNA (Fig. 2A, *right panel*). When epithelial cells (MDBK and m-ICcL2) were pre-treated overnight with SB203580 (25 μM) and then washed, this led to a decrease of about 30% in the number of infected cells in both cell lines. This decrease was reversed by adding anisomycin (1 μg/ml), an agonist of the p38 MAPK, to SB203580 pre-treated cells during infection (Fig. 2B) suggesting that the inhibitory effect on host cell invasion by *E*. *tenella* results only partially from the host p38 MAPK inhibition. Previous work showed that the ability of *T*. *gondii* to infect cells increased as cells proceeded from G1 phase to the S phase of their growth cycle and decreased as cells entered G2M [26,27]. Indeed, as the host p38 MAPK play a role in cell cycle regulation [24], SB203580 may modify the frequency of the different cell cycle phases and subsequently cell invasion. The host cell cycle was therefore analyzed after SB203580 treatment. Epithelial cells (MDBK, m-ICcL2 and CLEC-213) were treated overnight with SB203580 (25 μM) but no change in cell cycle phase was measured in our experimental conditions (Fig. 2C, S3 Fig. panel B). The 20–34% decrease in cell invasion observed during host p38 MAPK inhibition is therefore not caused by a change in host cell cycle in m-ICcL2 and MDBK. When chicken epithelial cells (CLEC-213) were pre-treated with SB203580 (25μM) overnight and washed, pretreatment did not modify host cell invasion (S3 Fig. panel A) leading to the hypothesis that this inhibitor might be less potent on chicken MAPK than on the mammalian p38 MAPK. These data suggest that the decrease in cell invasion in the presence of SB203580 is mostly due to a direct effect on the parasite itself which would be consistent with the higher IC50 found with the chicken cell line compared to the mammalian cell lines. Overall, these data suggest that the inhibitor may have a variable effect on the host cells depending on species but may affect mostly parasite p38 MAPK homologues. Although there is a very high amino acid identity of the p38 MAPK between chicken and mouse or bovine species (94%), a region located at the amino acids 236–257 presents poor homology (S3 Fig. panel C) and could contribute to variable effect of SB203580 pretreatment on cell invasion depending on the cell line used. This hypothesis would need to be further investigated.

### *E*. *tenella* possesses putative p38α MAPK homologues

We next investigated if the decrease in cell invasion resulted also from a direct effect of the inhibitor SB203580 on the parasite. We first sought for p38α MAPK homologues in the *E*. *tenella* database (www.toxodb.org). Blast of *Mus musculus* p38α MAPK (MAPK14; NM_001168508) protein on *E*. *tenella* protein database led to the identification of *E*. *tenella* putative kinases (accession number ETH_00035055, ETH_00005380, ETH_00028715, ETH_00004310, ETH_00004180) presenting 32–42% identity and that are mainly Cyclin dependent like kinases (CDKs), MAPK, Glycogen synthase kinases, CDK-like kinases (CMGC kinases, Table 1). Multiple sequence alignment of these kinases clearly revealed the eukaryotic protein kinase catalytic domain described by Hanks (2003) [17] (Table 2). The D168 of the motif Asp-Phe-Gly (DFG, subdomain VII) and the K53 of the motif VAXK (subdomain II) of the mouse p38α MAPK is present in all the parasite kinase homologues. Moreover, in the mouse p38α MAPK, T180 and Y182 are exposed on the surface of the 3D protein and need to be phosphorylated to turn the p38 MAPK into the activated form [28]. Both of these amino acids are found in the parasite kinases ETH_00004180 and ETH_00035055. Overall, we found a high conservation between motifs of the mouse p38α MAPK and parasite GMGC kinases.

**Table 1:** p38α MAPK homologues in *E*. *tenella*.

Proteins	% identity	Properties	Score	e values
ETH_00035055	37% (9–341AA)	CMGC kinase, MAPK family TgMAPK2 putative,598 AA	253	9e-80
ETH_00005380	34%(142–502 AA)		234	1e-72
ETH_00004180	42%(118–272; 354–549 AA)		152	5e-41
ETH_00028715	32%(64–354 AA)	CMGC kinase, MAPK family, putative, 551 AA	152	6e-43
ETH_00004310	38%(2–210 AA)	CMGC kinase, MAPK family (ERK) TgMAPK-1, putative, 1125 AA	150	3e-44

The mouse p38α MAPK was blasted on the *E*. *tenella* databases (toxodb.org). The first five homologue kinase proteins with the lowest e values are represented in the table.

**Table 2:** Alignment of the first five *E*. *tenella* homologue kinase proteins to the mouse p38α MAPK.

Proteins	Eukaryotic protein kinase catalytic domain				
	Subdomains				
Homologues	I GXGX[*FY*]GXV	II VAXK^53^	VIb HRDLKPXN	VII D^168^FGLAR	VIII T^180^XY^182^TXXYXAPE
NM_00116850 *Mus musculus* p38α MAPK	**G**S**G**A*Y* **G**S**V**	*V* **A**V**KK**	**HRD**L**KP**S**N**	**DFG** *L* **A** *R*	*T* *G *Y* * *V*A*TRW* **YR** *A* **P** *E*
ETH_00035055	**G**K**G** *AY* **G**V**V**	*V* **A**L**KK**	**HRD**M**KP**S**N**	**DFG** *L* **A** *R*	*T* *D *Y* * *V*A*TRW* **YR** *A* **P** *E*
ETH_00005380	**G**T**G**S*Y* **G**H**V**	*V* **A**I**KK**	**HRD**L**KP**A**N**	**DFG** *L* **A** *R*	*T* *GHVV*TRW* **YR** *A* **P**E
ETH_00004180	**G**S**G**A*Y* **G**C**V**	*V* **A**V**KK**	**HRD**L**KP**S**N**	**DFG** *L* **A** *R*	*T* *D *Y* * *V*V*TRW* **YR**P*PE*
ETH_00028175	**G**N**G**SF**G**V**V**	*V* **A**I**KK**	**HRD**V**KP**Q**N**	**DFG**S**A**K	VA*Y* *ICS*R*F**YR** *A* **P** *E*
ETH_00004310	**G**E**G**T*Y* **G**V**V**	C**A**L**KK**	**HRD**L**KP**Q**N**	**DFG** *L* **A** *R*	*T* *HE *V*V*T*L*W* **YR** *A* **P**D

* AA phosphorylated.

The first five *E. tenella* homologue kinase proteins with the lowest e values (Table 1) are aligned to the mouse p38α MAPK. The main motifs of subdomains of the eukaryotic protein kinase catalytic domain of the mammalian p38α MAPK are aligned to the five parasite kinases. In bold, the common amino acids are shown; in italic are amino acids found at a high frequency among proteins analyzed. The amino acids K53 and D168 (numbered in *Mus musculus*) are both required for catalytic activity. The amino acids T180 and Y182 (numbered in *Mus musculus*) are exposed at the surface of the inactivated p38α MAPK and phosphorylated by MAP kinases kinases leading to the activation of the p38α MAPK.

SB203580 is a pyridinylimidazole p38 MAPK inhibitor and binds to the ATP site of the p38 MAPK [29]. The pyrrole 4-[2-(4-fluorophenyl)-5-(1-methylpiperidine-4-yl)-1H-pyrrol-3-yl] pyridine which possesses a similar structure to that of SB203580 was found to inhibit cGMP-dependent protein kinase (PKG) from Apicomplexa [16,30,31]. Alignment of the mouse p38α MAPK with the EtPKG (ETH_00017800) reveals 21.6% identity and the presence of the protein kinase catalytic domain (S1 Table) with important amino acids for the kinase catalytic activity. The amino acids K53 and D168 are conserved in the EtPKG while T180 and Y182 in mouse PKG are found to be Y and T (S1 Table). Alignment of EtPKG with other Apicomplexa (*T*. *gondii* and *P*. *falciparum*) revealed 64–72% identity (S1 Table). These data suggest that SB203580 may not only interact with the host p38α MAPK but also with other parasite kinases such as CMGC kinases and PKG.

### SB203580 decreases parasite motility

**Fig 3:**
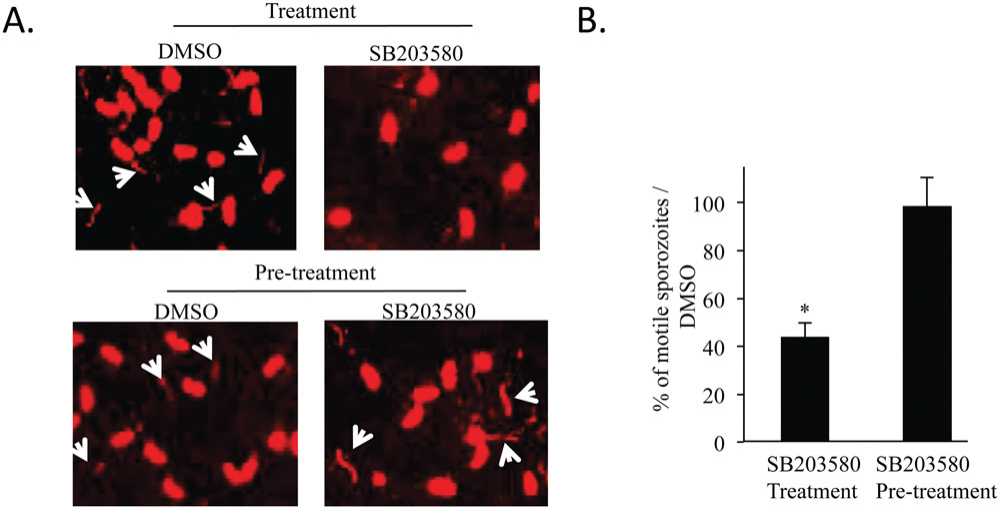
SB203580 decreases parasite motility. (**A**) Effect of SB203580 on sporozoite trail deposits during parasite gliding. Treatment: sporozoites placed on glass slides covered with gelatin were incubated for 1 h in the presence of SB203580 (25 μM) or DMSO. Pre-treatment: Sporozoites were first treated with SB203580 (25 μM) or DMSO for 1 h; after chasing, the sporozoite suspension was placed on glass slides for 1 h. After fixation, deposited molecules forming trails were revealed by immunofluorescence using a mouse polyclonal antibody against *E*. *tenella* sporozoites. Pictures are representative of 3 independent experiments. (**B**) Effect of SB203580 on sporozoite motility. Sporozoites were treated or pre-treated as described in (**A**). In this experiment, sporozoite gliding motility was assessed by videorecording and quantified by counting motile sporozoites over a period of 30 s. Data represent the mean of 6 independent experiments ± SEM. Statistical significance was calculated using the Student t test: *P < 0.05.

As we identified putative parasite kinase homologues to the mammalian p38α MAPK, we sought for some direct effect of SB203580 on the parasite by using a host cell free system [32]. Here, we determined parasite motility by two different methods that led to the same conclusion. We first stained trails of parasite proteins left on glass surfaces (Fig. 3A) with a mouse polyclonal antibody against *E*. *tenella* sporozoites. Secondly, we counted motile sporozoites by videomicroscopy (Fig. 3B). In both experiments, SB203580 induced a strong inhibitory effect on parasite gliding motility; however, this inhibitory effect is quickly reversible after chasing the inhibitor. Altogether, those data clearly show a decrease in parasite motility in the presence of SB203580 suggesting a direct effect of this inhibitor on the parasite.

### SB203580 decreases micronemal protein secretion

**Fig 4:**
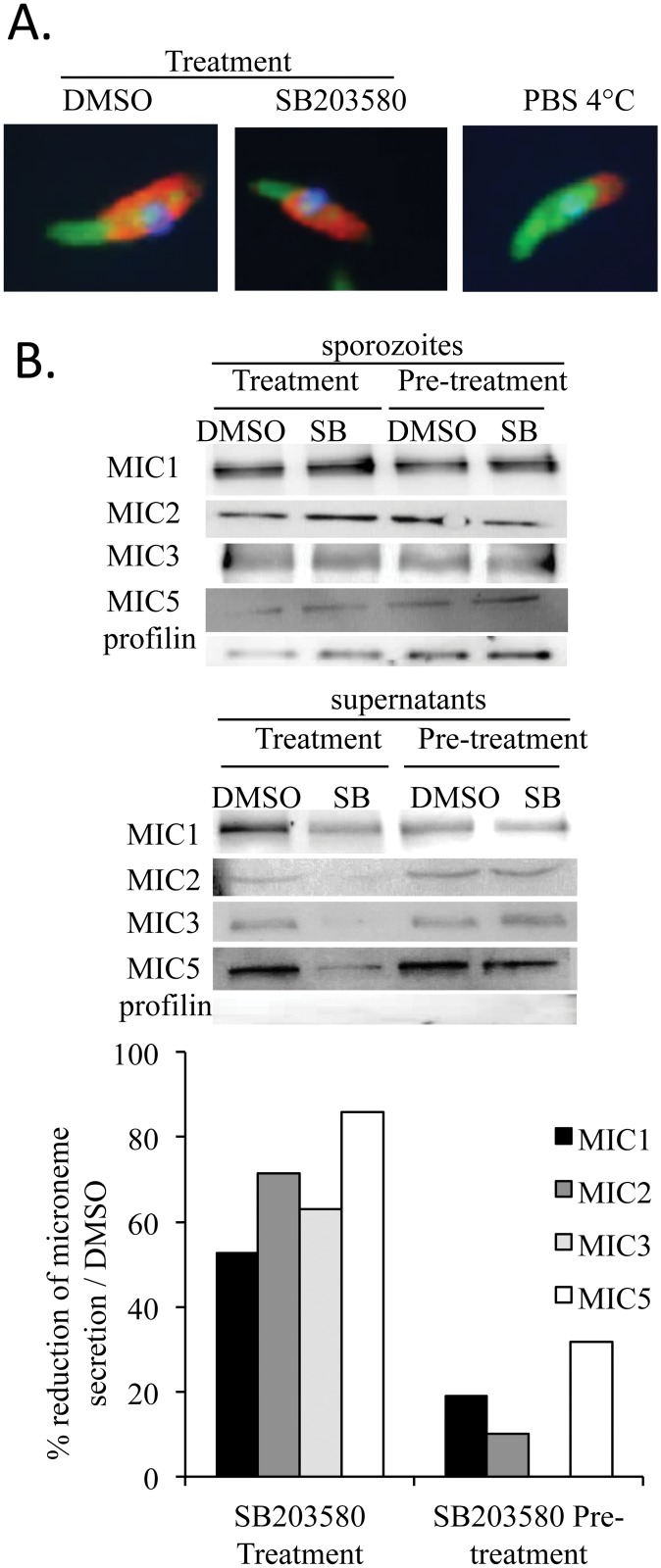
SB203580 decreases parasite micronemal protein secretion. Treatment consisted in incubation of sporozoites for 2 h in complete cell culture medium with SB203580 (25 μM) or DMSO. Pre-treatment consisted in first incubating sporozoites with SB203580 (25 μM) or DMSO for 1 h. Then, after chasing, sporozoites were incubated in complete cell culture medium for 2 h. After incubation, sporozoites were fixed for detection of micronemal proteins onto the surface of the parasite by immunofluorescence (**A**) or centrifuged for detecting micronemal proteins secreted in the medium and present in sporozoites (pellet) by western blotting (**B**). (**A**) Effect of SB203580 on micronemal protein distribution onto the surface *E*. *tenella*. This was determined by immunofluorescence. Sporozoites in PBS at 4°C were used as a negative control. (**B**) Effect of SB203580 on micronemal protein secretion. This was evaluated by western blotting in the supernatants and parasite lysates. Profilin was used as a control of the number of sporozoites. Quantification of secreted micronemal proteins in supernatants was performed using Bio-Profil Bio-1D++ software and is represented by histograms compared to the micronemal protein content in sporozoites. Data are representative of 2 experiments.

Invasion of epithelial cells is accompanied by the secretion of proteins from specialized organelles, micronemes and redistribution of these proteins onto the surface of the parasite [10,11,32–34]. To study the secretion and the translocation of micronemal proteins onto the parasite surface during gliding and invasion, we used a host cell free system previously described by Bumstead and Tomley (2000; [32]) in which the induction of micronemal protein secretion is stimulated by exposing parasites to albumin or FBS. Parasites were incubated in cell culture medium supplemented with 5% FBS and subsequently immunostained with mAb anti-EtMIC2. With or without treatment with SB203580, EtMIC2 was localized on the two third of the parasite surface (Fig. 4A). As expected, a negative control showed that in the absence of FBS, EtMIC2 stayed at the apical site of the parasite. Observations were similar for all the micronemal proteins analyzed (EtMIC1, EtMIC3 and EtMIC5). Then, we studied micronemal protein secretion by western blotting. Profilin an actin binding protein was used as an internal control of parasite load. Treatment of sporozoites with SB203580 (25 μM) led to a significant decrease in all the micronemal protein secretion. However, when sporozoites were pre-treated with SB203580 or DMSO and then incubated in complete cell culture medium containing a concentration of the inhibitor lower than 1 μM, micronemal protein secretion was totally restored suggesting that the inhibitory effect of SB203580 on parasite p38 MAPK homologues was quickly reversible (Fig. 4B). These data suggest that, when parasite micronemal protein secretion is triggered by contact with the host cell, SB203580 decreases this secretion by inhibiting parasite p38 MAPK homologues. Moreover, this inhibitor has no effect on micronemal protein redistribution onto the parasite surface meaning that parasite kinase homologues to the p38 MAPK are not implicated in this process.

### SB203580 does not permanently alter parasite invasion

**Fig 5:**
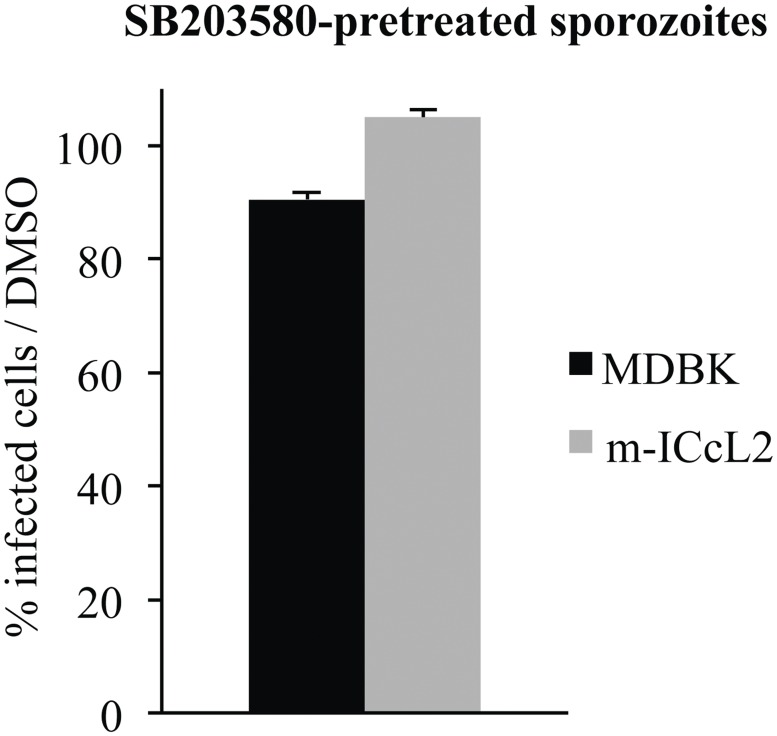
SB203580 does not permanently alter parasite invasion. Sporozoites were incubated for 1 h with SB203580 (25 μM); then, after chasing, sporozoites were incubated with cells for 2 h. Cell invasion was monitored and is represented as the percentage of infected cells compared to DMSO pre-treated-sporozoites. Data represent the mean of 3–4 experiments ± SEM.

As shown in Figs. 3 and 4B, gliding motility and micronemal protein secretion are altered in sporozoites incubated in the presence of SB203580 (25 μM) while these parasite functions are restored when the inhibitor is chased. These data are correlated with a recovery of cell invasion by SB203580 pre-treated parasites after a chase of the inhibitor suggesting a reversibility of this inhibitor on sporozoite kinase homologues (Fig. 5). Overall, these results show a direct effect of SB203580 on parasite p38 MAPK homologues resulting in a decrease in parasite motility and micronemal protein secretion and subsequently in cell invasion.

## Discussion

Protein kinases known to play an important role in proliferation, differentiation and pathogenesis of apicomplexan parasites are attractive targets for chemotherapy [35,36]. In various parasitic infections, kinase inhibitors targeting the protein kinase C have been shown to decrease host cell infection [20,37–39]. In a few studies related to cell invasion, inhibition of the p38 MAPK and ERK pathways blocked cell invasion by *T*. *gondii* [20,22] but the mechanism remains to be identified.

Here, we focused on the implication of kinases in the entry of the apicomplexan parasite *Eimeria tenella* into epithelial cells. In our study, we used a wide range of inhibitor concentrations to carefully determine the doses that were not cytotoxic for both epithelial cell and parasite. In these optimized conditions, the inhibitors of p38 MAPK (SB203580) and JNKII (SP600125) pathways decreased cell invasion by *E*. *tenella* in a dose dependent manner (IC50 for SB203580 is 9–11.5–23 μM). Moreover, *in vivo* studies demonstrated that administration of p38 MAPK inhibitors increased mouse survival and decreased *T*. *gondii* replication [18,19] and that infection of JNKII knockout mice led to host resistance by lowering *T*. *gondii* parasite burden and therefore increasing host survival [21]. Altogether, the p38 MAPK inhibitor is a common inhibitor for both *T*. *gondii* and *E*. *tenella* leading to a decrease in cell invasion. As the ERK pathway inhibitor blocked cell entry of *T*. *gondii* [20,22] and has no effect on *E*. *tenella* entry in our experimental conditions, this suggests that cell invasion mechanism for these parasites involves similar but also different set of kinases.

As we observed a stronger inhibitory effect on cell invasion with SB203580, we chose to focus on the p38 MAPK inhibitor for the rest of our study. In our first experiments, cells and parasites were both in contact with the inhibitor; we then sought for the respective implication of the host p38α MAPK and parasite putative p38α MAPK homologues in the invasion process. Specific inhibition of the host p38α MAPK using a siRNA approach led only to a 20% decrease in cell invasion by *E*. *tenella*. These data were confirmed when cells were pre-treated with the inhibitor SB203580 and then treated during infection with anisomycin; therefore, these data suggest only a minor role of the host p38 MAPK in the entry of the parasite. Similarly, specific inhibition of host p38 MAPK with recombinant adenoviruses expressing dominant negative MKK3 or MKK6 has minimal effect (~20% inhibition) on intracellular *T*. *gondii* replication [19]. The role of the host p38 MAPK in cell invasion could be linked to its role in many cellular functions such as cell cycle, metabolism, apoptosis, survival, differentiation, migration and cytoskeleton [40]. We then explored if the SB203580 affected the host cell cycle which could have contributed to changes in host cell permissivity as described for *Toxoplasma gondii* [26,27]. However, as we used confluent cells, SB203580 treatment did not significantly modified host cell cycle suggesting that the 20–34% decrease in mammalian cell invasion is not due to a change in the host cell cycle. While it is well known that invasion is an active process of the parasite, several studies pointed out that the entry of the Apicomplexa also requires rearrangements of the host cytoskeleton [41–44]. In the presence of SB203580, limited rearrangement of the host cytoskeleton due to a reduced phosphorylation activity of the p38 MAPK may alter the entry of parasite into the host cells. Further studies would be needed to explore the role of the p38 MAPK on the host cytoskeleton when the parasite *E*. *tenella* enters epithelial cells. However, few experiments performed with a chicken epithelial cell line suggest that the SB203580 may mostly act on parasite kinases as pretreating chicken epithelial cells with this inhibitor did not modify host cell invasion by *Eimeria tenella*.

In Apicomplexa parasites, several kinases have been described [45] and play an important role in parasite development and virulence [46–50]. Then, as the effect of the host cell in parasite entry was determined to be about 20–34%, we hypothesized that the major effect of the inhibitor SB203580 could be on the parasite itself by acting on parasite p38 MAPK homologues or closely related kinases. We then sought for p38α MAPK homologues in the *E*. *tenella* database and identified five *E*. *tenella* kinases (Table 1) with 32–42% amino acids identity and possessing, as for the p38 MAPK, an ATP binding site that could be targeted by the SB203580 inhibitor.

Entry of parasites into host epithelial cells is powered by parasite gliding motility and depends on the distribution onto the parasite surface of proteins from specialized organelles, micronemes, and secretion of their protein content [11,33,34]. The signaling pathways implicated in parasite gliding motility and micronemal protein secretion are poorly understood, but transient calcium intracellular increase is known to be essential [10,51]. In *T*. *gondii*, parasite motility and micronemal protein secretion are also regulated by kinases: protein kinase C [10], cyclic GMP-dependent protein kinase [16,30,31] and calcium-dependent protein kinase (CDPK) 1 [52]. In *E*. *tenella*, Han et al. (2013) [53] demonstrated recently that the inhibition of EtCDPK3 using inhibitory antibodies against this kinase led to a decrease in cell invasion. In this context, we studied the effect of SB203580 on parasite motility, micronemal protein distribution onto the parasite surface and micronemal protein secretion. A decrease in motility and micronemal protein secretion (EtMIC1, 2, 3 and 5) was observed in the presence of SB203580. After chasing the inhibitor, motility and micronemal protein secretion were restored to their initial level. The reversibility of the parasite motility and micronemal protein secretion after removal of the inhibitor is well correlated with the restoration of cell invasion. These data suggest that parasite p38 MAPK homologues are involved in both parasite gliding motility and micronemal protein secretion. However, in our experimental conditions, parasite kinases were not involved in micronemal distribution onto the parasite surface. In previous studies, it was reported that micronemal protein secretion is not coupled to actinomyosin motor while parasite motility and micronemal protein translocation are actin-dependent [32,54]. Then, as the mechanism of micronemal protein secretion and parasite gliding motility are different, we hypothesized that SB203580 may inhibit one or several parasite p38 MAPK homologues implicated in either micronemal protein secretion or motility, or both. Moreover, following apical secretion, micronemal protein present onto the parasite surface are proteolytically processed during invasion involving micronemal protein proteases that cleave micronemal proteins in their transmembrane domain leading to their release (for review [55]). As proteolysis of micronemal proteins was shown to be essential for releasing cell surface adhesins prior to cell entry by apicomplexan parasites [56], we propose that, if p38 MAPK homologues regulate these proteases, SB203580 would indirectly inhibit micronemal protein release from the surface of the first invasive stage of the parasite.

Overall, these data suggest that SB203580 acts directly on several parasite kinases to inhibit parasite motility, micronemal protein secretion and subsequently cell invasion. The generation of new anticoccidial drugs is eagerly awaited to control chicken coccidiosis. Further experiments will therefore be necessary to identify precisely the parasite kinase(s) implicated and subsequent development of new highly specific compounds against this/these target(s).

## Materials and Methods

### Ethic statements

Experimental protocols were conducted in compliance with French legislation (Décret: 2001–464 29/05/01) and EEC regulations (86/609/CEE) governing the care and use of laboratory animals, after validation by the local ethics committee for animal experimentation (Comité d’Ethique pour l’Expérimentation Animale Val de Loire, CEEA VdL): 2012–11–09.

### Parasite and epithelial cell lines

Group of outbred PA12 chickens of 4 to 6 weeks old age were infected orally with 10^4^ sporulated oocysts of *E*. *tenella* Wisconsin strain expressing constitutively the yellow fluorescent protein (YFP). Sporozoites were transfected in our lab with a plasmid carrying the YFP gene under the control of *E*. *tenella mic1* promoter as described by [57]. Seven days post-inoculation, the chickens were sacrificed and unsporulated oocysts were harvested from infected caeca. Oocysts were purified and sporulated at 26°C for 72 h as previously described [58]. Sporozoites were obtained after breaking sporulated oocysts with glass beads and incubating sporocysts with excystation medium (0.5% biliary salts, 0.25% trypsin in PBS, pH 7.4) at 41°C for 1 h. After purification on cotton and on polycarbonate filters (5 μm; GE Water & Process Technologies), sporozoites were counted on Thoma cell and ready to use for experiments. Madin-Darby bovine kidney (MDBK) cells were grown in HAM’s F12 medium (Lonza) containing 5% fetal bovine serum (FBS; Lonza), 2 mM glutamine (Lonza), 10 UI/ml penicillin and 10 μg/ml streptomycin (Lonza) [59]. Transimmortalized mouse intestinal epithelial cell (m-ICcL2) were grown in DMEM/HAM’s F12 (Gibco) supplemented with 5% FBS, 1% non-essential amino acids (Gibco), 5 μg/ml human transferrin, 50 nM dexamethasone, 1 nM triiodothyronine, 10 ng/ml epidermal growth factor (Sigma), 30 nM sodium selenite (Sigma), 5 μg/ml insulin (Sigma), 10 UI/ml penicillin and 10 μg/ml streptomycin as described by Bens et al., (1996) [60]. Epithelial cells were trypsinized for cell passage and experiments. Cells were counted on Malassez cell and 2 × 10^5^ cells were plated in 24-well plate for viability and invasion assays and 10^6^ cells were plated in 6-well plate for cell cycle experiments.

### Epithelial cell and sporozoite viability

Epithelial cell viability was assessed by 3-(4,5-Dimethyl-2-thiazolyl)-2,5-diphenyl-2H-tetrazolium bromide (MTT, Sigma) assay after 24 h incubation with PD98059, SP600125, SB203580 (Calbiochem) or DMSO (Sigma) at 37°C. Then, cells were incubated for 4 h with MTT that is metabolized to formazan by intact mitochondrial dehydrogenases. Formazan crystals are solubilized with acidified isopropanol and the intensity is measured spectrophotometrically at 570 nm with background subtraction at 690 nM. Cell viability is represented as the % of viability in inhibitor-treated conditions (PD98059, SP600125, SB203580) compared to DMSO-treated conditions. Sporozoite viability was assessed by Evans blue exclusion assay (0.1 mg/ml; RAL Diagnostic) and by carboxyfluorescein diacetate, succinimidyl ester (CFDA-SE)-propidium iodide (PI) assay (8 μg/ml CFDA-SE, 5 μg/ml PI; Molecular Probes) after incubation with PD98059, SP600125, SB203580 or DMSO for 2 h at 37°C. Sporozoites were visualized by fluorescent microscopy (Olympus BX41 microscope). Evans blue penetrates the sporozoite when membranes are damaged. In the Evans blue exclusion assay, total number of sporozoites was evaluated in bright light and dead sporozoites were detected in red in fluorescent light. In CFDA-SE-PI assay, CFDA-SE is metabolized by active mitochondria and then fluoresces green. Propidium iodide penetrates cells and binds to DNA when the cell membrane integrity is compromised leading to a red fluorescence of sporozoites. Parasite viability was then assessed by counting the number of green (viable) and red (dead) cells. The % of parasite viability corresponds to the number of viable parasites / total number of parasites in inhibitor-treated conditions compared to the number of viable parasites / total number of parasites in DMSO-treated conditions.

### Invasion assay

For invasion assay, 2×10^5^ epithelial cells (MDBK or m-ICcL2) were placed on glass coverslip at the bottom of 24-well plates. For treatment, cells and parasites were cocultured in the presence of SB203580 at concentrations (0–25 μM) or anisomycin (1 μg/ml; Sigma). For host cell pre-treatments, epithelial cells were pre-treated overnight with SB203580 (25 μM) or DMSO and washed before infection. For parasite pre-treatments, sporozoites were pre-treated for 1 h with SB203580 (25 μM) or DMSO in PBS and then, the inhibitor was chased to a concentration lower then 1 μM. After washing or chasing the inhibitor, infection was performed using purified sporozoites (4×10^5^) for 2 h at 37°C, 5% CO_2_ in complete cell culture medium. After 2 h coculture, cells were washed and fixed with paraformaldehyde 4%. Monolayers were mounted in Vectashield containing 1.5 μg/ml DAPI (Clinisciences) to label nuclei. The percentage of infected cells was determined compared to the total number of cells. For each coverslips, at least three different microscope fields were captured and more than 200 cells were counted for each condition in at least three independent experiments. Values are standardized to DMSO and are reported as mean ± standard errors.

### Gene silencing and RT-qPCR

m-ICcL2 (70000 cells) were transfected overnight with a mixture of four siRNA for p38 MAPK (Mn_Mapk14_2, Mn_Mapk14_3, Mn_Mapk14_4, Mn_Mapk14_5; 2.5 nM each; Qiagen) and interferin as described in manufacturer’s instruction (Polyplus). Scrambled control siRNA that had no sequence homology to any known genes was used as negative (non silencing) control siRNA. For invasion assay, transfected cells were infected with 350000 parasites for 2 h at 37°C and processed as described above. Values are standardized to scramble control siRNA and are reported as mean ± standard errors. Gene silencing was confirmed by RT-qPCR. Cells were lysed in TRIzol (Life Technologies) and RNA was extracted with 20% chloroform (Sigma), precipitated with 50% isopropanol (Sigma) and resuspended in nuclease-free water. Total RNA (1 μg) was used to reverse transcribe total mRNA using an oligo(dT)15 primer (Promega) and reverse transcriptase II (Invitrogen). Segments of cDNAs were amplified using specific primers to mp38 MAPK 5′-ATCATTCACGCCAAAAGGAC-3′ and 5′-AGCTTCTGGCACTTCACGAT-3′ and to three housekeeping genes: peptidylpropyl isomerase A (PPIA) 5′-GTCTCCTTCGAGCTGTTTGC-3′ and 5′–AGCATACAGGTCCTGGCATC-3′; TATA-binding protein (TBP) 5′-CAGCCTTCCACCTTATGCTC-3′ and 5′-TTGCTGCTGCTGTCTTTGTT-3′; Hypoxanthine phosphoribosyl transferase (HPRT) 5′-TGCTCGAGATGTCATGAAGG-3′ and 5′-TATGTCCCCCGTTGACTGAT-3′ (Eurogentec). Real-time RT-qPCRs were run on a Bio-Rad Chromo4 (Bio-Rad). Results were normalized to the three reference genes. Mean of gene expression values are expressed as relative values to scramble samples.

### Cell cycle

For cell cycle experiments, 10^6^ epithelial cells (MDBK or m-ICcL2) were placed in a 6-well plate. First, cells were treated overnight with SB203580 (25 μM). Cells were trypsinized, washed and fixed with 70% cold ethanol. After fixation, cells were treated with RNases (0.2 mg/ml, Sigma) for 1 h at 37°C, stained with propidium iodide (50 μg/ml) and analyzed by flow cytometry (Moflo Beckman Coulter, Fort Collins, Colorado, USA). The percentage of cells in each phase was assessed by using the software MultiCycle for windows Phoenix flow system, Inc. San-Diego, CA, USA.

### Gliding motility, micronemal protein translocation and micronemal protein secretion assays

To study gliding motility, micronemal protein translocation onto the parasite surface and the release micronemal proteins, we used a host cell free system as previously described by Bumstead and Tomley, 2000 [32]. When parasite were treated, sporozoites were incubated at 37°C in DMEM/HAM’s F12 supplemented with 5% FBS in the presence of SB203580 (25 μM) or DMSO for 1–2 h. When parasite were pre-treated, sporozoites were first incubated with SB203580 (25 μM) or DMSO in PBS for 1 h at room temperature; then, the inhibitor was chased with DMEM/HAM’s F12 supplemented with 5% FBS to a concentration of inhibitor less than 1 μM and sporozoites were incubated at 37°C for 1–2 h. Sporozoite incubations were performed at the same time for pre-treated and treated parasites. After incubation, parasite gliding motility, micronemal protein distribution onto the parasite surface and micronemal protein secretion assays were performed.

For gliding motility assay using immunofluorescence to reveal protein deposits on the surface of a glass slide, coverslips covered with gelatin 0.2% were placed at the bottom of a 24-well plate. Parasites were allowed to glide for 1 h at 37°C in complete cell culture medium and then fixed with paraformaldehyde 4%. Sporozoite protein deposits were detected by using a mouse polyclonal antibody anti-*E*.*tenella* sporozoite lysate (1/100) and an anti-mouse conjugated to Alexa 594 (1/1000; Invitrogen). For gliding motility assay using videomicroscopy, sporozoites were placed on a 24-well plate in the experimental conditions for 1 h and parasite motility was recorded for 30 s (Optika Vision Lite 2.1). Motile sporozoites were counted and represented by a histogram. For studying micronemal protein distribution onto the parasite surface, parasites were placed on a coverslip for 2 h in complete cell culture medium and fixed with paraformaldehyde 4% (Diapath). Micronemal protein repartition onto the surface of sporozoites was detected by using different antibodies: anti-EtMIC1 (1/1000; chicken), anti-EtMIC2 (1/1000; rabbit), anti-EtMIC3 (1/1000; mouse), anti-EtMIC5 (1/1000; rabbit) and the corresponding secondary antibodies conjugated to Alexa 594 (1/1000). Parasites were visualized by fluorescent microscopy (Zeiss Axiovert 200 microscope, Carl Zeiss, Germany). For parasite micronemal protein secretion assay, sporozoites (2x10^6^) were incubated for 2 h in complete cell culture medium and centrifuged; sporozoite pellets and supernatants were collected. Sporozoites were lysed in RIPA buffer supplemented with protease inhibitors (AEBSF 0.1 mM, EDTA 0.15 mM, pepstatin 1 mg/ml, E-64 11 μM; Sigma) and sonicated (amplitude 30, 3 s for 15 s; Vibracell 75455, Bioblock Scientific, USA). Supernatants and sporozoite lysates were mixed with loading buffer, and heated at 96°C for 5 min then electrophoresed on 8% SDS-PAGE minigels and transferred to nitrocellulose membrane (GE Healthcare) for 1 h by wet electroblotting. Membranes were blocked for 1 h in 5% milk powder (w/v) in PBS. Micronemal proteins were detected using anti-EtMIC1 (1/1000; chicken; 75 KDa), anti-EtMIC2 (1/1000; rabbit, 35 KDa), anti-EtMIC3 (1/1000; mouse, 109 KDa), anti-EtMIC5 (1/1000; rabbit, 100 KDa) antibodies and the corresponding secondary antibodies conjugated to horseradish peroxidase (1/1000; Sigma). An antiserum specific for profilin (1/1000, rabbit, 19 KDa [61]) was used as internal control of parasite load and negative control in the supernatant. Immunoblots were revealed by SuperSignal West Pico chemiluminescent substrates (Pierce) and detected using a ChemiSmart5000 (Vilber Lourmat, France).

## Supporting Information

S1 Fig Evaluation of the toxicity of the p38 MAPK kinase inhibitor, SB203580 on mammalian epithelial cell lines. Epithelial cells viability was measured by MTT after 24 h incubation with SB203580 for both m-ICcL2 (**Figure A**) and MDBK (**Figure B**).
(TIF)

S2 Fig Dose response curve of p38 MAPK inhibitor on chicken lung epithelial cell (CLEC-213) invasion. (**Panel A**) Evaluation of the toxicity of the p38 MAPK kinase inhibitor, SB203580 on the chicken epithelial cell line, CLEC-213. The cell line was maintained as described by Esnault et al 2011 [25]. Epithelial cells viability was measured by MTT after 2 h incubation with SB203580. (Panel **B**) Dose response curve of the p38 MAPK inhibitor, SB203580, on epithelial cell invasion. Cell invasion is represented as percentage of infected cells compared to DMSO treated cells.
(TIF)

S3 Fig Effect of SB203580 pre-treatment of epithelial cell on cell invasion. (**Panel A**) Effect of SB203580 on epithelial cell cycle. CLEC-213 were treated overnight with SB203580 (25 μM) or DMSO. After washing, cells were fixed, stained with propidium iodide and the epithelial cell cycle was assessed by flow cytometry. Data represent the mean of 2 experiments ± SEM. (**Panel B**) Pre-treatment: epithelial cells (CLEC-213) were incubated overnight with either SB203580 (25 μM) or DMSO. After pre-treatment, cells were washed and infected. *Left panel*: Epithelial cells viability was measured by MTT after overnight incubation with SB203580, 25μM. Data are represented as percentage of viable cells compared to DMSO pre-treated cells. *Right panel*: Cell invasion is represented as percentage of infected cells compared to DMSO pre-treated cells. (**Panel C**) Amino acid comparison of chicken mouse and bovine p38 MAPK in the region of amino acids 236–257. In red, the common amino acids are shown; in blue are amino acids found at a high frequency among proteins analyzed; in black are divergent amino acids.
(TIF)

S1 Table Alignment of EtPKG, TgPKG and PfPKG to the mammalian p38α MAPK. In bold, the common amino acids are shown; in italic are amino acids found at a high frequency among protein analyzed. The amino acids K53 and D168 (numbered in *Mus musculus*) are both required for catalytic activity. The amino acids T180 and Y182 (numbered in *Mus musculus*) are exposed at the surface of the inactivated p38α MAPK and phosphorylated by MAP kinases kinases leading to the activation of the p38α MAPK.
(TIF)

S1 Video Videorecording of parasite in the presence of DMSO. Sporozoites were incubated for 1 h in the presence of DMSO and videorecording was performed over a period of 30 s.
(MOV)

S2 Video Videorecording of parasite in the presence of SB203580. Sporozoites were incubated for 1 h in the presence of SB203580 (25 μM) and videorecording was performed over a period of 30 s.
(MOV)

S3 Video Videorecording of parasite pre-treated with DMSO. Sporozoites were first treated with DMSO for 1 h; after chasing, the sporozoite suspension was incubated for 1 h and videorecording was performed over a period of 30 s.
(MOV)

S4 Video Videorecording of parasite pre-treated with SB203580. Sporozoites were first treated with SB203580 (25 μM) for 1 h; after chasing, the sporozoite suspension was incubated for 1 h and videorecording was performed over a period of 30 s.
(MOV)
