# Supplementary figures and images for: Reduced Parasite Motility and Micronemal Protein Secretion by a p38 MAPK Inhibitor Leads to a Severe Impairment of Cell Invasion by the Apicomplexan Parasite Eimeria tenella
Source: PLoS One. 2015 Feb 17;10(2):e0116509. doi: 10.1371/journal.pone.0116509 (PMC4331428; doi:10.1371/journal.pone.0116509)

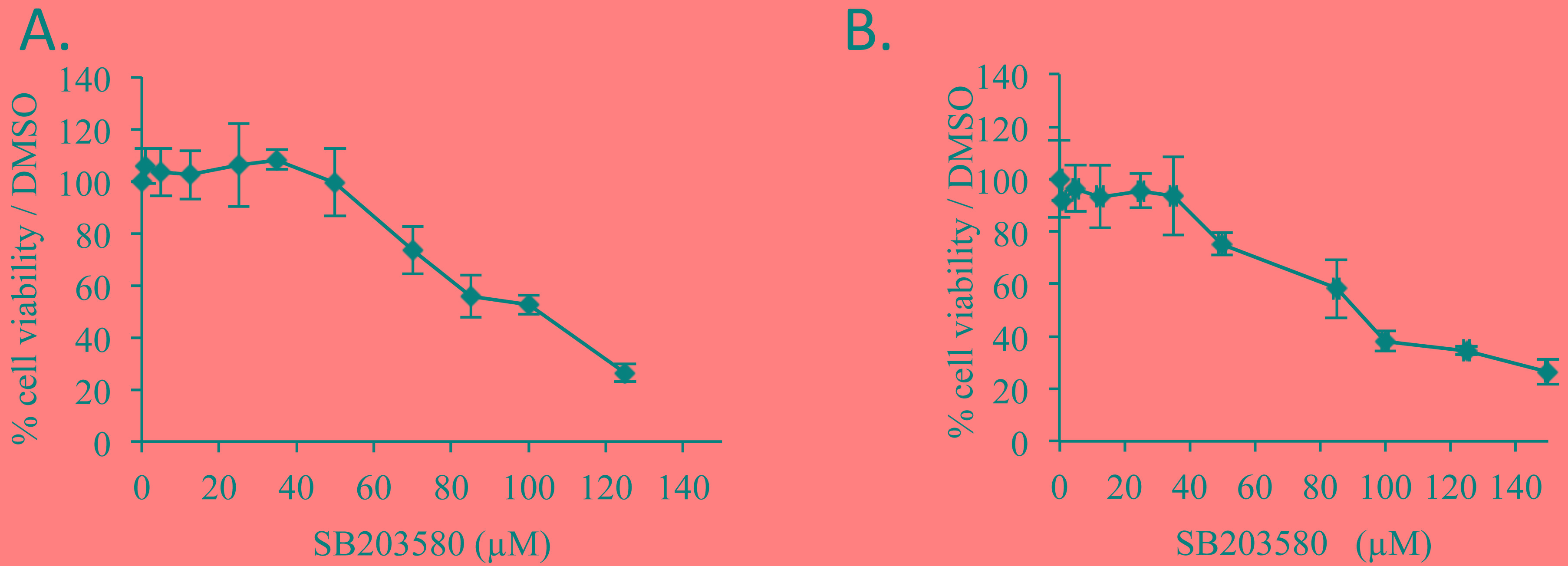

Supplement: S1 Fig — Epithelial cells viability was measured by MTT after 24 h incubation with SB203580 for both m-ICcL2 (Figure A) and MDBK (Figure B). (TIF) [file pone.0116509.s001.tif]

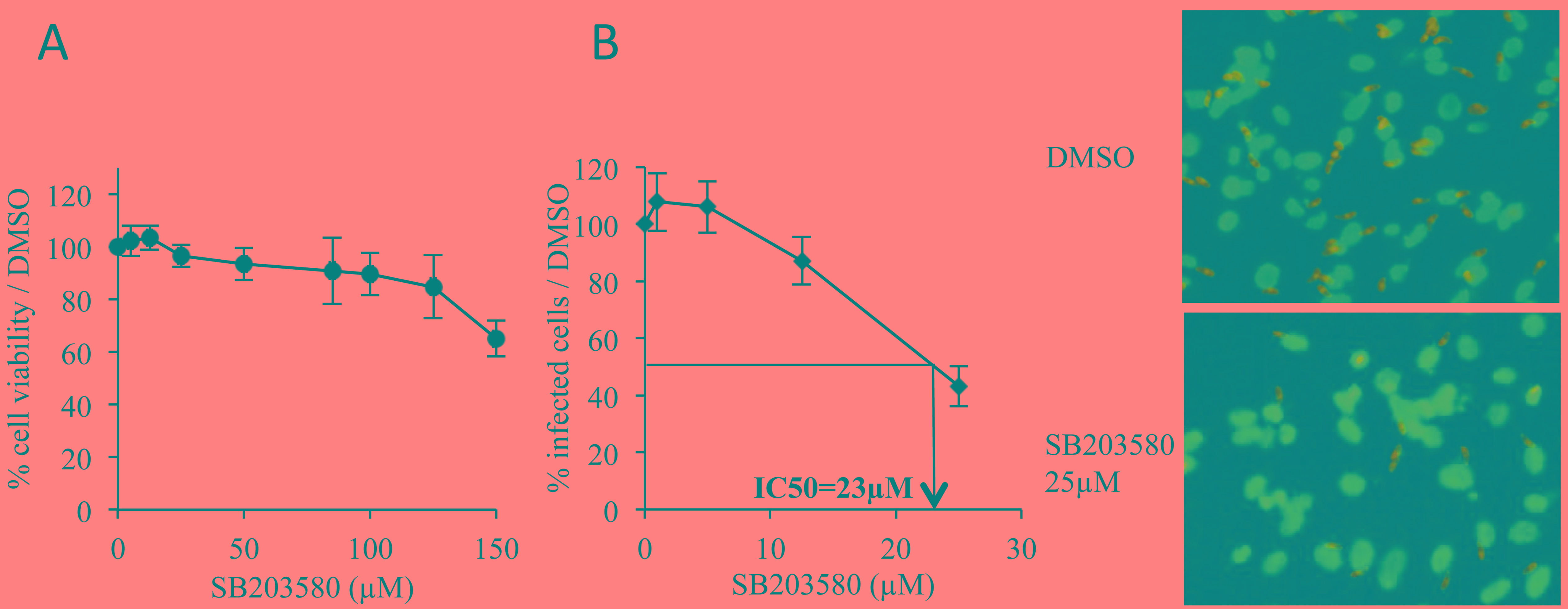

Supplement: S2 Fig — (Panel A) Evaluation of the toxicity of the p38 MAPK kinase inhibitor, SB203580 on the chicken epithelial cell line, CLEC-213. The cell line was maintained as described by Esnault et al 2011 [25]. Epithelial cells viability was measured by MTT after 2 h incubation with SB203580. (Panel B) Dose response curve of the p38 MAPK inhibitor, SB203580, on epithelial cell invasion. Cell invasion is represented as percentage of infected cells compared to DMSO treated cells. (TIF) [file pone.0116509.s002.tif]

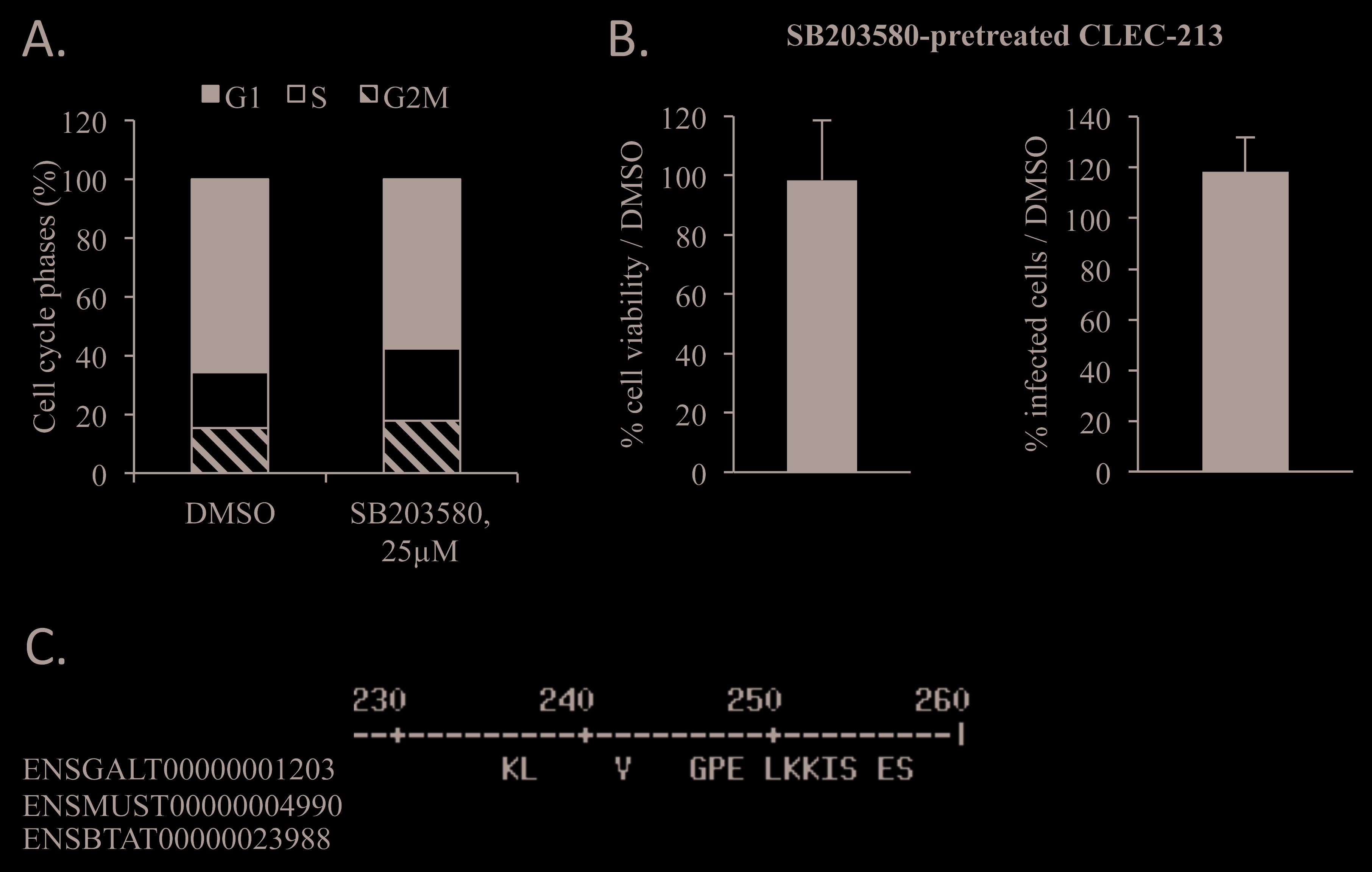

Supplement: S3 Fig — (Panel A) Effect of SB203580 on epithelial cell cycle. CLEC-213 were treated overnight with SB203580 (25 μM) or DMSO. After washing, cells were fixed, stained with propidium iodide and the epithelial cell cycle was assessed by flow cytometry. Data represent the mean of 2 experiments ± SEM. (Panel B) Pre-treatment: epithelial cells (CLEC-213) were incubated overnight with either SB203580 (25 μM) or DMSO. After pre-treatment, cells were washed and infected. Left panel: Epithelial cells viability was measured by MTT after overnight incubation with SB203580, 25μM. Data are represented as percentage of viable cells compared to DMSO pre-treated cells. Right panel: Cell invasion is represented as percentage of infected cells compared to DMSO pre-treated cells. (Panel C) Amino acid comparison of chicken mouse and bovine p38 MAPK in the region of amino acids 236–257. In red, the common amino acids are shown; in blue are amino acids found at a high frequency among proteins analyzed; in black are divergent amino acids. (TIF) [file pone.0116509.s003.tif]

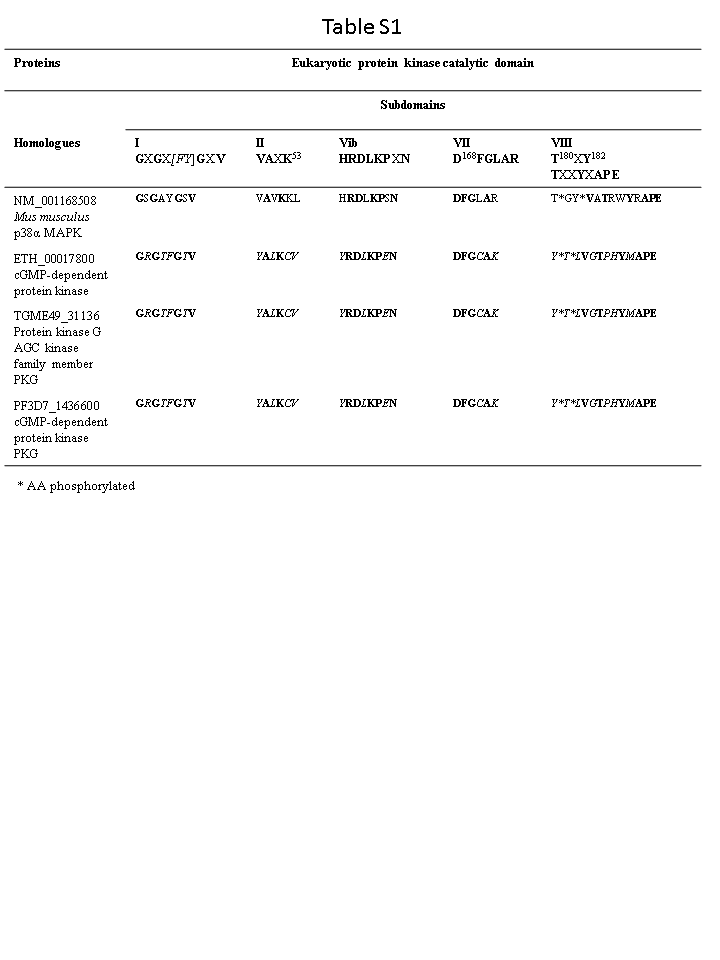

Supplement: S1 Table — In bold, the common amino acids are shown; in italic are amino acids found at a high frequency among protein analyzed. The amino acids K53 and D168 (numbered in Mus musculus) are both required for catalytic activity. The amino acids T180 and Y182 (numbered in Mus musculus) are exposed at the surface of the inactivated p38α MAPK and phosphorylated by MAP kinases kinases leading to the activation of the p38α MAPK. (TIF) [file pone.0116509.s004.tif]
